# Educational Attainment and Self-Rated Oral Health among American Older Adults: Hispanics’ Diminished Returns

**DOI:** 10.3390/dj7040097

**Published:** 2019-10-01

**Authors:** Shervin Assari, Mohsen Bazargan

**Affiliations:** 1 Department of Family Medicine, Charles R. Drew University of Medicine and Science, Los Angeles, CA 90059, USA; Mohsenbazargan@cdrewu.edu; 2 Department of Family Medicine, University of California Los Angeles (UCLA), Los Angeles, CA 90095, USA

**Keywords:** Hispanics, oral health, educational attainment, socioeconomic status, socioeconomic position

## Abstract

*Background*: Minorities’ diminished returns (MDRs) refer to systemically weaker effects of socioeconomic status (SES), particularly educational attainment, on the health of non-Whites compared to Whites. *Aim*: Using a nationally representative sample, we aimed to investigate ethnic differences in the effect of SES (educational attainment) on the self-rated oral health of Hispanic older adults in the US. *Methods*: This study analyzed the University of Michigan National Poll on Healthy Aging (UM-NPHA) 2017 data, which included 2131 older adults who were 50 to 80 years old (202 Hispanics and 1929 non-Hispanics). Ethnicity, race, educational attainment (SES), age, gender, employment, retirement, and self-rated oral health (single item) were measured. Logistic regressions were applied for data analysis. *Results*: High educational attainment was associated with lower odds of poor oral health in the pooled sample, net of all covariates. The effect of educational attainment on poor self-rated oral health was found to be weaker for Hispanics than for non-Hispanics. *Conclusion*: We observed MDRs of educational attainment (SES) on oral health for Hispanic older adults. In other words, compared to non-Hispanics, Hispanics gain less oral health from their educational attainment (SES).

## 1. Background

Ethnic and economic disparities in oral health are well established in the US [1,2]. Hispanics have a higher risk of poor oral health than non-Hispanics. At least some of these ethnic differences in oral health can be attributed to low socioeconomic status (SES), which translates to reduced access to dental care [3]. As a result, SES is believed to explain some of the disparities in oral health across ethnic minority groups [1,2,3].

Socioeconomic status, particularly high educational attainment, is generally protective against poor oral health and promotes access to dental services [1,2,3]. Among adults 35 to 44 years old, those with less than a high school education are three times more likely to have untreated tooth decay than those with at least some college education [4]. The same holds for destructive periodontal (gum) disease.

Various mechanisms are involved in explaining the protective effects of SES against poor health across domains including oral health [5,6]. First, educational attainment and income are associated with tooth brushing and flossing [7,8,9,10,11,12], behaviors that promote oral health [7,13,14,15,16]. Second, highly educated, employed individuals have better access to dental care because, in the US, employment is the main gatekeeper to the health care system [3]. In addition, high SES is associated with higher oral health literacy and seeing oral health as a part of overall health [17,18,19,20]. Some barriers that limit the chance of having a dental visit for individuals with low SES are lack of insurance, lack of transportation, and having an inflexible schedule [21].

Minorities’ diminished returns (MDRs) refer to the smaller health effects of SES, particularly educational attainment, for members of the minority group, compared to Whites [22,23]. Unfortunately, MDRs (smaller health gains on the part of non-Whites from their SES resources) are systemically overlooked as a cause of inequalities in health [22,23]. While alternative explanations propose high stress, discrimination, the bias of the health care system, poor health care access, and low SES as mediators of disparities, MDRs focuses on the minorities’ diminished returns of SES [22,23]. 

### Aim

This nationally representative study of older Americans tested ethnic variations in the effects of educational attainment on self-rated oral health. Building on the MDRs theory, we expected education to be less strongly associated with oral health for Hispanics than for non-Hispanics. 

## 2. Methods

### 2.1. Study Design and Setting

Data came from the University of Michigan National Poll on Healthy Aging (UM-NPHA, 2017). The UM-NPHA is an online survey of older adults in the US. The study uses a nationally representative sample. Conducted by the University of Michigan (UM) Institute for Healthcare Policy and Innovation, UM-NPHA aims to monitor changes in the health and well-being of older Americans.

The UM-NPHA used the Knowledge Networks framework for sampling. This was an online internet panel with a nationally representative sample of US adults. The UM-NPHA gathered data on the health and wellbeing of American adults who are at least 50 years old. Using random sampling, the NPHA has provided an opportunity to study the combined effects of race/ethnicity, gender, and SES on the daily life of older adults in the US. The study collected data on demographics, SES indicators, social networks, and health care use. 

### 2.2. Analytical Sample for This Analysis

The current study included Hispanics and non-Hispanics. Exclusion criteria for the UM-NPHA were (1) being younger than 50 years and (2) being institutionalized.

### 2.3. Ethics

This study was a secondary analysis of fully de-identified publicly available data from the UM-NPHA. According to the National Institutes of Health definition of human subjects’ research (https://grants.nih.gov/policy/humansubjects/hs-decision.htm), the current study did not involve human subjects and was therefore exempt from the review of the study protocol by the ethical review board. All UM-NPHA participants signed informed consent.

### 2.4. Study Measures

Study variables included ethnicity, race, age, gender, employment, retirement, income, education, and self-rated oral health.

#### 2.4.1. The Independent Variable

*Educational Attainment.* Educational attainment was measured as a continuous measure with a potential range from 1 to 14. Responses included: No formal education (1) to a professional or doctorate degree (14). A higher score meant higher education attainment.

#### 2.4.2. Moderating Variables

*Ethnicity.* Ethnicity was the moderator in this study. Ethnicity, a self-identified variable, was treated as a dichotomous measure (non-Hispanics 0 [the reference group], Hispanics 1). Ethnicity refers to groups based on heritage, culture, country of origin, customs, beliefs, and norms.

#### 2.4.3. Covariate Variables

*Demographic Variables.* Gender and age were the study covariates. Age was treated as a continuous variable (years since birth). Gender (male 1, female 0) was treated as a dichotomous measure.

*Race.* Self-identified race was a covariate. Race was operationalized as a dichotomous variable (Black 1, any other race 0). Race refers to groups based on skin color, hair texture, and other aspects of appearance.

*Employment Status.* Employment status was operationalized as a dichotomous variable (any other status 0 versus full-time or part-time employed 1).

*Retirement*. Retirement was a dichotomous variable (not retired 0 versus retired 1).

*Income.* Income was a dichotomous variable: 0) Less than $30,000 versus 1) $30,000 or more. 

#### 2.4.4. The Dependent Variable

*Self-Rated Oral Health.* Self-Rated Oral Health was measured using a single item: “How do you rate the health of your teeth and gums?” Responses included “Excellent/Very Good”, “Good”, or “Fair/Poor”. We coded “fair/poor” oral health as 1 and “excellent/very good/good” as 0.

### 2.5. Statistical Analysis

Data analysis was performed using SPSS 23.0 (IBM Support, Among, NY, USA). To describe the sample overall, we reported the frequency (%) and mean (Standard deviation). We used bivariate correlation tests to study the bivariate correlations between all the study variables. We then ran four logistic regression models. *Model 1* only included the main effects of ethnicity, educational attainment, and covariates. *Model 2* included the main effects as well as an interaction term between ethnicity and educational attainment. *Model 3* and *Model 4* were conducted in each ethnic group. Odds ratios (OR), B, standard errors (SE), 95% CIs, and *p*-values were reported.

## 3. Results

### 3.1. Descriptive Statistics

Table 1 shows the descriptive statistics. The table shows that the mean age of the participants was 64 years, with an SD of eight years. From all participants, 27.2% reported poor oral health. This rate was higher in Hispanics (34.0%) than non-Hispanics (26.5%) (*p* < 0.05, Chi square).

### 3.2. Bivariate Correlations

Table 2 shows a summary of the bivariate correlations in the overall sample. Hispanic ethnicity was negatively correlated with age, retirement, and education, and positively correlated with poor oral health. Poor oral health was negatively correlated with income and educational attainment, and employment but positively correlated with retirement. (Table 2)

### 3.3. Overall Model

As shown in Table 3, *Model 1* only included the main effects. *Model 2* also included ethnicity by the interaction term of educational attainment. *Model 1* documented an inverse association between the level of educational attainment and odds of poor self-rated oral health that was above and beyond all the study covariates. *Model 2* showed a statistically significant interaction between the effects of ethnicity and educational attainment on the odds of poor oral health, indicating smaller protective effects of educational attainment against poor oral health for Hispanics in comparison with non-Hispanics.

### 3.4. Ethnic-Specific Models

Table 4 shows the results of two logistic regression models by ethnicity. In all models, educational attainment was the independent variable, poor oral health was the dependent variable, and the covariates were controlled. *Model 3* and *Model 4* were performed in non-Hispanics and Hispanics, respectively.

*Model 3* showed an inverse association between educational attainment and odds of poor self-rated oral health above and beyond the covariates in non-Hispanics. *Model 4* did not show a significant protective effect for high educational attainment against the odds of poor oral health for Hispanics.

## 4. Discussion

The current study revealed two main findings. First, we found a negative association between high educational attainment and poor self-rated oral health. Second, we observed that the protective effects of high educational attainment against poor self-rated oral health are greater in non-Hispanic older adults than in Hispanic older adults.

Our first finding is in line with the literature, which has frequently shown a protective effect of SES against poor oral health [1,10,11,12]. The effects of SES on oral health may be due to the presence of multiple mechanisms, such as access to the oral health care services through health insurance, pro-oral health behaviors, and enhanced access to oral health care services due to the fact of having insurance [10,11,12]. This main effect of educational attainment is nothing new. We already know that having some college education, for example, is associated with a three-fold decrease in the odds of untreated tooth decay, compared to individuals who only have a high school education. Similarly, a college education is associated with a decline in the chances of having destructive periodontal (gum) disease [4]. 

We are aware of at least two other studies on MDRs of SES indicators on the oral health of racial and ethnic minorities relative to non-Hispanic Whites. In the first study, which used data from the Collaborative Psychiatric Epidemiology Surveys (2003), MDRs were observed: the positive effects of educational attainment, household income, employment, and family type on self-rated oral health were less for Hispanics than for non-Hispanics. In other words, while high educational attainment, household income, being employed, and being married were associated with better self-rated oral health, these effects were stronger for non-Hispanics than for Hispanics [1]. In the second study, MDRs were found for effects on family income on the risk of unmet dental care needs in Black children compared to White children. Data from the National Survey of Children’s Health showed that unmet dental care need was a function of income for White children but not for Black children [3]. That is, SES seems to translate to increased oral health for Whites but not for racial and ethnic minorities. But none of these studies were on older adults [1]. The unique contribution of this study is to extend such results to Hispanic older adults.

The result of this study is in support of MDRs among Hispanic older adults. Less is known about MDRs among Hispanics than among Blacks. Previous research has shown Blacks’ MDRs for the effects of educational attainment on depression [24], obesity [25,26,27], self-rated health [28,29], and mortality [30,31]. Blacks’ MDRs are also not specific to educational attainment [32,33,34] and are shown for income [24,26,35], employment [30], and marital status [36]. The same patterns are seen in children [26,35,37,38], adults [39,40,41], and older adults [42]. However, much more is known about MDRs in Blacks [26,35,43] than Hispanics [1] and other marginalized groups [25]. Although these MDRs are more pronounced for Blacks and are seen across SES resources, outcomes, and age groups, they may also apply to Hispanics. 

The results suggest a mechanism behind ethnic oral health disparities. Not all racial/ethnic health disparities are due to the SES that covaries with the minority status. This study showed that ethnic minority individuals remain at risk for poor oral health, even when they are highly educated. This means diminished returns of SES, particularly education, are also among the mechanisms by which ethnicity is linked to oral health.

This study exclusively focused on diminished returns of educational attainment as the main SES indicator, but income is another important SES factor. Income is a main cause of oral health disparities [44]. Poverty reduces the chance of receiving dental care [4]. In the year 2010, 42% and 70% of people above and below the federal poverty line reported a dental visit in the last year [45]. Two out of ten low-income people do not have any dental visits over five years [45]. More research is needed on diminished returns of various SES indicators.

## 5. Limitations

The current study measured oral health via self-report. This is a standard measure that has been well described. Survey respondents were asked to rate their oral health from 1 to 5, with a higher score indicating worse perceived oral health. Survey respondents did not receive any specific instruction on how they should rate their oral health. Numerous factors had the potential to confound self-reported oral health. For example, cultural norms and oral health literacy affect self-assessment of oral health. Self-rated oral health may not reflect objective measures across groups of people, as only some people may report poor oral health in the presence of dental and oral health problems such as decay, tooth loss, toothache, and chronic periodontitis. Self-rated oral health may even reflect cosmetic issues for high SES individuals. Finally, self-rated oral health may differently reflect objective oral problems based on individuals’ oral health literacy and awareness. All these issues provide opportunities for future research on MDRs of SES on objective and subjective oral health measures across marginalized people. 

We lacked considerable knowledge of the Hispanic participants’ country of origin, nativity, and immigration status. The US-born Hispanics and immigrant Hispanics may differ in their educational attainment and in their ability to leverage their education to gain employment and access to health care. Although compared to Non-Hispanics, Hispanics had slightly less educational attainment and income, they were well educated and mostly employed. Future research may compare MDRs for educational attainment not only based on ethnicity but also immigration status, nativity, and country of origin. Education may operate differently for Mexicans, Cubans, Puerto Ricans, and other subgroups of Hispanic populations. Cultural norms, behaviors, and societal treatment all impact oral health yet differ widely across subgroups of Hispanic people. 

Many good, generic, patient-reported dental outcome measures exist today [46]. Such measures may or may not replicate the findings reported here. There is a need to explore the MDRs of SES on objective measures of oral health that also have high validity. Such research will determine if MDRs only apply to subjective measures of oral health or if they are also relevant for objective oral health measures. 

This study used an income cutoff of $30,000. This approach was taken because we needed a common threshold independent of place and geographic location. This was because we were interested in national rather than local economic policies and programs that can mitigate the MDRs of SES. However, we understand that poverty cutoffs differ in the South, Midwest, and East Coast. In addition, income above the poverty cutoff may not necessarily indicate the presence of sufficient income to pay for dental care. Also, Table 2 suggested only a modest correlation between income and education (*r* = 0.33), which indicated that higher education did not have a very strong impact on generating higher income and, probably, in greater access to money to pay for dental care in this population as well. Future research may test the results using more sensitive or specific cutoffs that enable a local definition of poverty. 

## 6. Conclusions

Major ethnic group differences were found in the effects of educational attainment on self-rated oral health. Among Hispanics, not all oral health disparities were due to the fact of a lower SES; some were due to the lower-than-expected returns on available SES. Compared to non-Hispanic Whites, Hispanics were at a relative disadvantage when it came to the oral health effects of SES resources, particularly educational attainment. We may see less-than-expected oral health in high-SES Hispanics. Future research should test how White privilege and marginalization contribute to these patterns.

## Figures and Tables

**Table 1:** Descriptive data of the pooled sample and in ethnic groups.

	All		Non-Hispanics		Hispanics	
	Mean	SD				
Age *^a^	64.10	7.98	64.38	7.98	61.44	7.49
Education (1–14) *^a^	10.36	1.96	10.47	1.87	9.28	2.45
Ethnicity						
Non-Hispanics	1929	90.5	1929	100.0	-	-
Hispanics	202	9.5	-	-	202	100.0
Race						
Non-Black	1943	91.2	1741	90.3	202	100.0
Black	188	8.8	188	9.7	0.0	0.0
Gender						
Female	1091	51.2	994	51.5	97	48.0
Male	1040	48.8	935	48.5	105	52.0
Retirement * ^b^						
No	1051	49.5	938	48.8	113	56.2
Yes	1074	50.5	986	51.2	88	43.8
Employed * ^b^						
No	1271	59.8	1161	60.3	110	54.7
Yes	854	40.2	763	39.7	91	45.3
Income * ^b^						
Less than $30,000	370	17.4	326	16.9	44	21.8
$30,000+	1761	82.6	1603	83.1	158	78.2
Self-Rated Oral Health * ^b^						
Good-Excellent	1547	72.8	1415	73.5	132	66.0
Poor-Fair	579	27.2	511	26.5	68	34.0

* *p* < 0.05. ^a^ Independent sample *t*-test. ^b^ Chi-square test.

**Table 2:** Spearman correlations in the pooled sample.

	1	2	3	4	5	6	7	8	9
1 Ethnicity (Hispanics)	1.00	−0.10 **	0.02	−0.11 **	−0.04 *	0.03	−0.04	−0.15 **	0.05 *
2 Race (Blacks)		1.00	0.01	−0.04 *	0.01	0.00	−0.10 **	−0.08 **	0.08 **
3 Gender (Male)			1.00	−0.01	−0.02	0.08 **	0.06 **	0.09 **	0.02
4 Age (Years)				1.00	0.64 **	−0.51 **	−0.02	0.03	0.01
5 Retirement					1.00	−0.83 **	−0.07 **	−0.03	0.05 *
6 Employed						1.00	0.17 **	0.11 **	−0.10 **
7 Income (≥$30,000)							1.00	0.33 **	−0.23 **
8 Education (1–14)								1.00	−0.23 **
9 Oral Health (Poor)									1.00

* *p* < 0.05, ** *p* < 0.01.

**Table 3:** Associations between educational attainment and poor oral health in the pooled sample.

	B	SE	OR = Exp (B)	95% CI for OR	*p*-Value
**Model 1 (Main Effects)**					
Ethnicity (Hispanics)	0.12	0.17	1.13	0.80–1.59	0.482
Race (Blacks)	0.36	0.17	1.44	1.03–2.01	0.035
Gender (Male)	0.21	0.10	1.24	1.01–1.51	0.041
Age (Years)	0.00	0.01	1.00	0.98–1.02	0.883
Retirement	−0.22	0.20	0.80	0.55–1.18	0.261
Employed	−0.50	0.18	0.61	0.42–0.86	0.006
Income (≥$30,000)	−0.77	0.13	0.46	0.36–0.60	< 0.001
Education (1–14)	−0.22	0.03	0.81	0.76–0.85	< 0.001
Constant	2.05	0.57	7.80		< 0.001
**Model 2 (M1 + Interactions)**					
Ethnicity (Hispanics)	−1.20	0.68	0.30	0.08–1.14	0.077
Race (Blacks)	0.35	0.17	1.42	1.01–1.99	0.042
Gender (Male)	0.22	0.10	1.24	1.02–1.53	0.035
Age (Years)	0.00	0.01	1.00	0.98–1.02	0.893
Retirement	−0.23	0.20	0.80	0.54–1.17	0.246
Employed	−0.51	0.18	0.60	0.42–0.86	0.005
Income (≥$30,000)	−0.76	0.13	0.47	0.36–0.60	< 0.001
Education (1–14)	−0.24	0.03	0.78	0.74–0.84	< 0.001
Education (Years) × Ethnicity	0.14	0.07	1.15	1.00–1.32	0.043
Constant	2.31	0.59	10.03		< 0.001

Outcome: Poor Self-Rated Oral Health. B = Regression Coefficient. CI = Confidence Interval. SE = Standard Errors. OR = Odds Ratio.

**Table 4:** Associations between educational attainment and poor oral health in the non-Hispanics and Hispanics.

	B	SE	OR = Exp(B)	95% CI for OR		*p*-Value
**Model 3 (Non-Hispanics)**						
Race (Blacks)	0.34	0.17	1.41	1.01–1.98		0.046
Gender (Male)	0.34	0.11	1.41	1.14–1.75		0.002
Age (Years)	−0.01	0.01	0.99	0.98–1.01		0.573
Retirement	−0.13	0.21	0.88	0.58–1.33		0.552
Employed	−0.50	0.20	0.61	0.41–0.89		0.011
Income (≥$30,000)	−0.73	0.14	0.48	0.37–0.64		< 0.001
Education (1–14)	−0.25	0.03	0.78	0.73–0.83		< 0.001
Constant	2.47	0.62	11.85			< 0.001
**Model 4 (Hispanics)**						
Race (Blacks)						
Gender (Male)	−0.77	0.32	0.47	0.25–0.87		0.017
Age (Years)	0.02	0.03	1.02	0.97–1.08		0.415
Retirement	−1.00	0.58	0.37	0.12–1.15		0.086
Employed	−0.59	0.52	0.55	0.20–1.52		0.252
Income (≥$30,000)	−1.08	0.39	0.34	0.16–0.73		0.005
Education (1–14)	−0.09	0.07	0.92	0.80–1.05		0.199
Constant	0.69	1.68	2.00			0.680

Outcome: Poor Self-Rated Oral Health. B = Regression Coefficient. CI = Confidence Interval. SE = Standard Error. OR = Odds Ratio.
